# Alpha Power Predicts Persistence of Bistable Perception

**DOI:** 10.1038/s41598-017-05610-8

**Published:** 2017-07-12

**Authors:** Giovanni Piantoni, Nico Romeijn, German Gomez-Herrero, Ysbrand D. Van Der Werf, Eus J. W. Van Someren

**Affiliations:** 1 Dept. Neurology, Massachusetts General Hospital, Harvard Medical School, Boston, MA USA; 2 Dept. Sleep and Cognition, Netherlands Institute for Neuroscience, Amsterdam, The Netherlands; 3 Dept. Anatomy and Neurosciences, VU University Medical Center, Amsterdam, The Netherlands; 4 Dept. Integrative Neurophysiology, VU University, Amsterdam, The Netherlands; 5 Dept. Psychiatry, VU University Medical Center, Amsterdam, The Netherlands

## Abstract

Perception is strongly affected by the intrinsic state of the brain, which controls the propensity to either maintain a particular perceptual interpretation or switch to another. To understand the mechanisms underlying the spontaneous drive of the brain to explore alternative interpretations of unchanging stimuli, we repeatedly recorded high-density EEG after normal sleep and after sleep deprivation while participants observed a Necker cube image and reported the durations of the alternating representations of their bistable perception. We found that local alpha power around the parieto-occipital sulcus within the first second after the emergence of a perceptual representation predicted the fate of its duration. An experimentally induced increase in alpha power by means of sleep deprivation increased the average duration of individual representations. Taken together, these findings show that high alpha power promotes the stability of a perceptual representation and suppresses switching to the alternative. The observations support the hypothesis that synchronization of alpha oscillations across a wide neuronal network promotes the maintenance and stabilization of its current perceptual representation. Elevated alpha power could also be key to the poorly understood cognitive deficits, that typically accompany sleep deprivation, such as the loss of mental flexibility and lapses of responsiveness.

## Introduction

An important feature of the perceptual system is its flexibility to process identical physical stimuli in different ways depending on the circumstances. It enables, for example, the maintenance of attention to stimulus properties with expected reward and prevents interference from distracting aspects of the same stimulus. This flexibility is highly dependent on and tightly controlled by ongoing brain activity^1, 2^. Even at rest, when faced with an unchanging physical stimulus, the brain continues to explore alternative perceptual interpretations, a property that becomes manifest when the stimulus is an ambiguous figure^3–5^.

There have been continued efforts to identify the neuronal correlates of this perceptual flexibility. One of the most likely candidates are the oscillations of neuronal origin, because the numerous combinations of frequency, amplitude, and phase modulation offer a sophisticated and precise mechanism to entrain diverse neuronal populations that encode the same perceptual object^6, 7^. The ability of oscillations, in particular alpha (~10 Hz), to encode multiple sources of information is demonstrated by the correlation of alpha power with the number of items in working memory^8^.

This process is thought to be mediated by the inhibition that alpha exerts on task-irrelevant brain regions, thereby favoring focused attention^9–12^. Spontaneous fluctuations in alpha power^13–16^ have been shown to determine the subject’s ability and accuracy to process stimuli^17–21^. These observations support the interpretation that alpha reflects a neural mechanism that regulates the amount of visual information that can be passed to higher-order areas, to conscious processing, and to the initiation of behavioral responses^11, 12, 22, 23^.

It remains an open question how fluctuations in alpha power affect the spontaneous changes in perception during bistable perception, because the level of the visual hierarchy at which alpha oscillations operate is a matter of controversy. If taken to work at a lower level, the inhibition associated with high alpha power should suppress the strength of both perceptual representations, resulting in faster perceptual alternations, similarly to the effect observed with stimuli of low contrast^24^. An alternative prediction is that inhibition occurs at the level of the competition between neural populations encoding the rivalling representations^25^, resulting in longer perceptual alternations.

These opposite predictions can be tested experimentally by measuring the degree to which alpha power affects the likelihood of a transition between two rivaling perceptual representations. Based on hypothesis that alpha plays an active role in stabilizing perception, we expect that its momentary amplitude will determine the degree of perceptual stability and that the spontaneous fluctuations in alpha power will affect the perceptual state. In order not to disturb this intrinsic rhythm by means of a stimulus-driven experimental paradigm, we took advantage of the spontaneous alternations in perception during a bistable perception with an unchanging stimulus. The spontaneous alternations between the perceptual representations provide a unique opportunity to investigate how neuronal dynamics modulate perception without changing stimulus properties according to a specific cognitive paradigm^4, 26, 27^.

## Results

**Figure 1:**
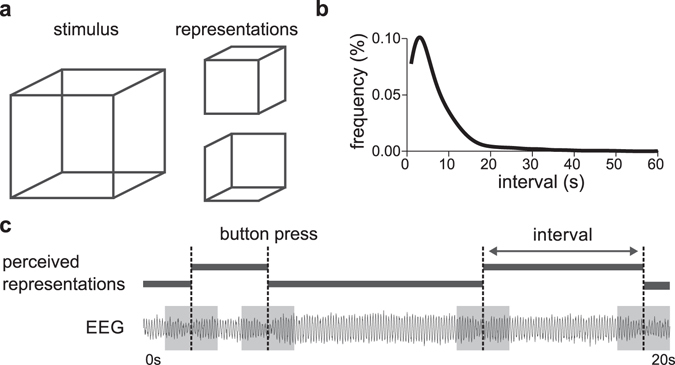
(**a**) The viewing of a Necker cube causes the perceptual system to alternate between two rivaling perceptual representations. The onset of each of the alternating percepts is reported by use of button presses. The perceptual duration of each individual percept is defined as the time interval between two button presses. (**b**) Probability distribution of perceptual durations of individual perceptual representations during Necker cube viewing after normal sleep. The distribution had a positive skew, with a mean value of 8.87 s and a median value of 5.94 s. (**c**) EEG data were recorded continuously while participants reported the perceived reversals of the representations of the ambiguous Necker cube by pressing a button. In the first analysis, alpha power was computed on whole perceptual durations, excluding the periods just preceding and following the button presses (gray areas). In a more finely grained second analysis, alpha power was calculated on a sliding window starting right after the emergence of a percept and evaluated on its predictive value for the perceptual duration of the present percept.

High-density electroencephalogram (EEG) was recorded five times a day for four minutes on two days in eight healthy participants, while they viewed a Necker cube image (Fig. 1a) and reported the alternation between the two bistable representations by pressing a button. The probability distribution of the perceptual durations showed the typical gamma distribution^4, 5, 28^ (Fig. 1b). Inward and outward representations of the Necker cube alternated in 91% of the reported percept emergence; 9% of the emerging percepts concerned return transitions^29^.

**Figure 2:**
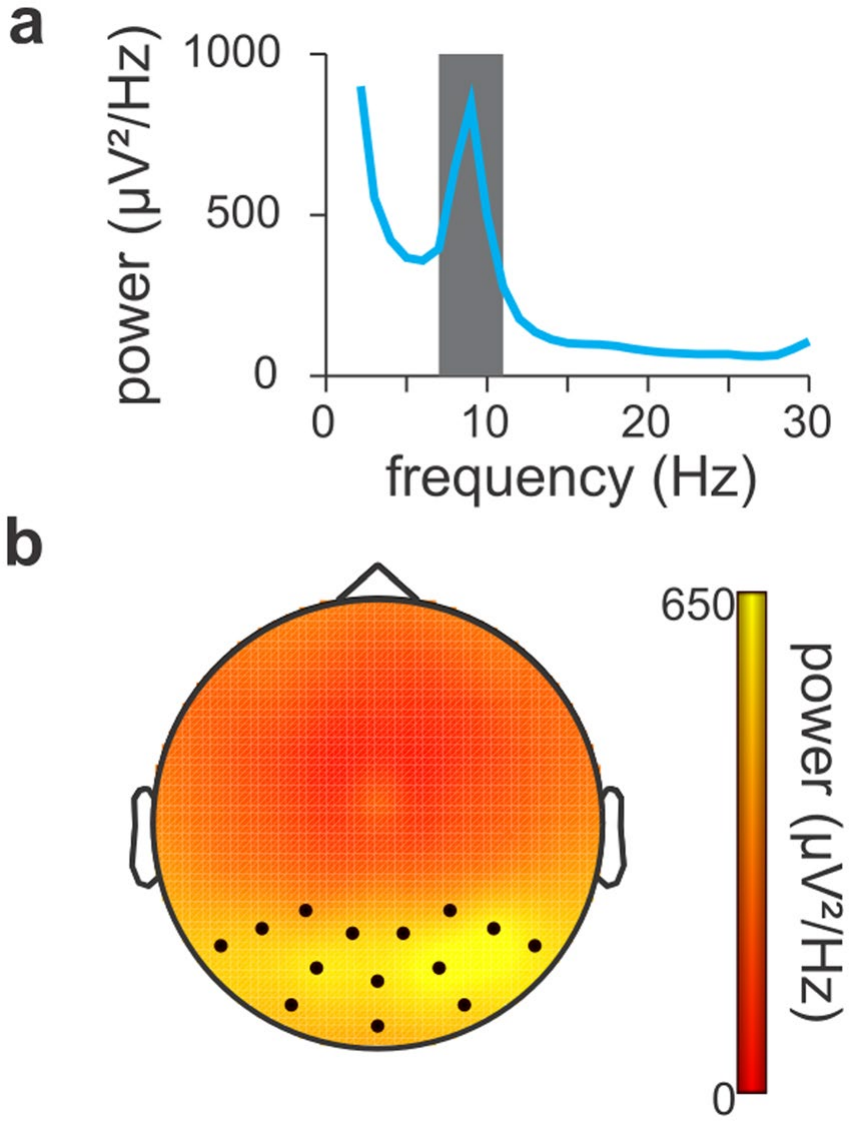
Distribution of alpha power after normal sleep in the frequency-, space-, and time-domain. (**a**) Power spectrum of the EEG recorded over parietal and occipital electrodes in the interval between the button presses indicating reversals. The power spectrum shows a clear peak at 9 Hz, the gray area indicates the frequency boundaries used in the subsequent analysis (7–11 Hz). (**b**) Topographic distribution of alpha power during periods of perceptual stability. For each stable period, alpha power was averaged over the parietal and occipital electrodes (indicated by a marker). The value was used as regressor to predict the perceptual duration of the present percept.

We first identified the frequency and spatial characteristics of alpha oscillations in our cohort (Fig. 2). Because alpha power peaked at 9 Hz with a characteristic posterior predominance, subsequent analysis procedures used the 7 to 11 Hz power band (gray bar in Fig. 2a) averaged over parietal and occipital electrodes (indicated by a marker in Fig. 2b). Analysis on the EEG recordings was computed in two ways: one analysis was conducted on the intervals between the perceptual reversals (indicated by the button presses) and another separate analysis was conducted on the periods around the perceptual reversals (Fig. 1c).

Average alpha power was calculated for each of the individually reported perceptual durations, using Welch’s method on 50% overlapping windows of 1 s duration, excluding the 2 s interval around the button press. Regression analysis revealed that more stable perceptual representations, i.e. of longer duration, were characterized by higher alpha power, while shorter lasting perceptual representations were characterized by lower alpha power (estimated effect of alpha power = 0.128 log(s)/log(µV^2^), s.e.m. = 0.039, *z*-value = 3.235, *p*-value < 0.001, *r*-value^2^ = 0.20).

**Figure 3:**
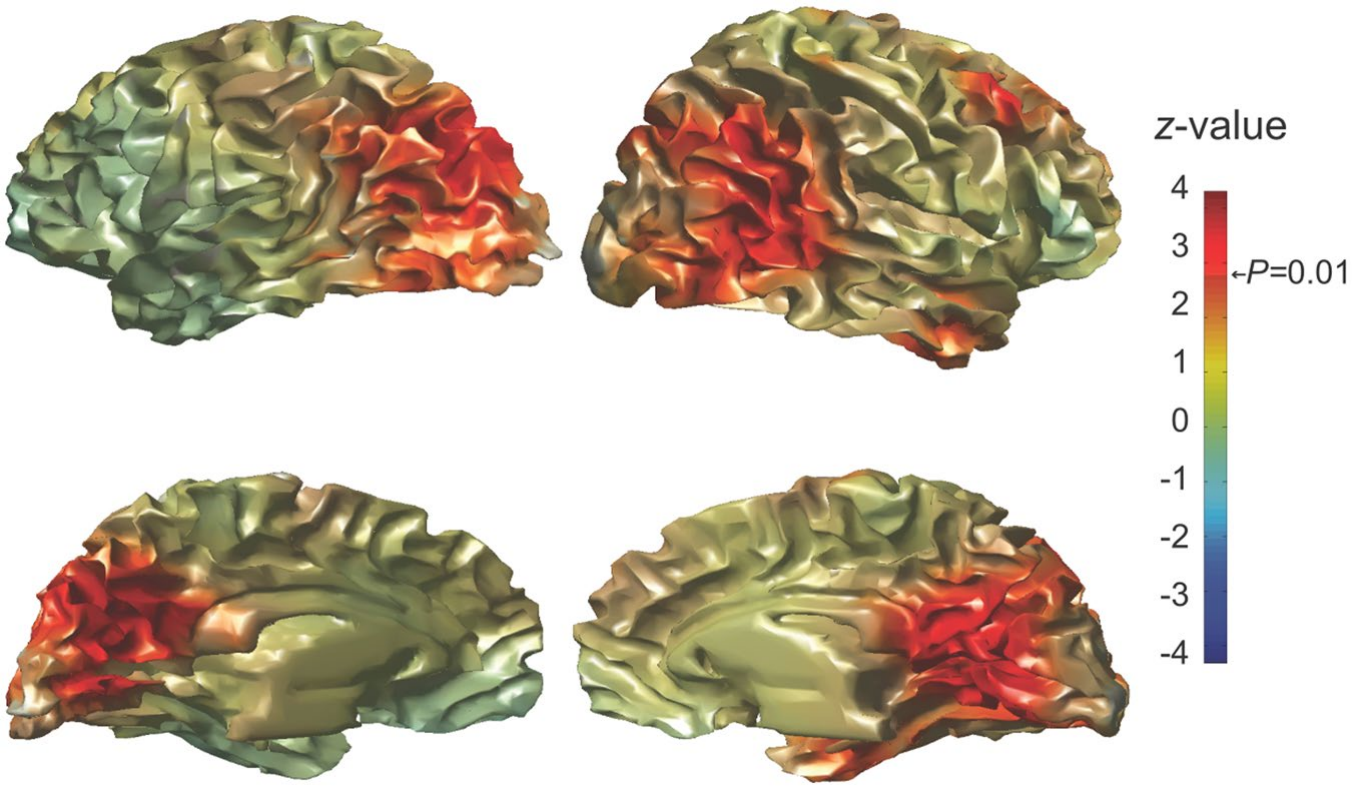
Beamformer-estimated voxels where alpha power correlated with the duration of individual perceptual representations. The estimated alpha power in each of the voxels was used as regressor of the perceptual duration, using a LMEM to return a *z*-value. The regression was calculated for each voxel of the brain on a 10 mm spaced grid of a template MRI, distributed with SPM8 and segmented^81^. For illustration purposes, the *z*-values are projected onto the cortical surface and the *z*-values are thresholded at 2.326, corresponding to a one-sided *p*-value = 0.01. The regions showing the strongest association are located in the cortical regions around the parieto-occipital sulcus.

Because alpha activity has been found to be highly localized across multiple cortical domains (prefrontal^30^, visual^17, 31^ and somatosensory^32^ cortices), we set to identify the local brain region that showed a significant correlation between alpha power and the duration of the individual perceptual representations. We first estimated alpha power in all the voxels that were inside the brain using a frequency-domain beamformer source-reconstruction method^33^ in the frequency band centered at 9 Hz with ± 2 Hz frequency smoothing^34^. The estimates of alpha power in each of 2015 voxels were used as explaining variables in a regression model of the duration of individual percepts. Figure 3 gives a graphical representation of the voxels where alpha power predicted the perceptual duration of a percept (*z*-value ≥ 2.326, one-sided *p*-value ≤ 0.01). The significant voxels were mostly localized in the bilateral medial parts of the superior occipital cortex and in the parieto-occipital junction.

**Figure 4:**
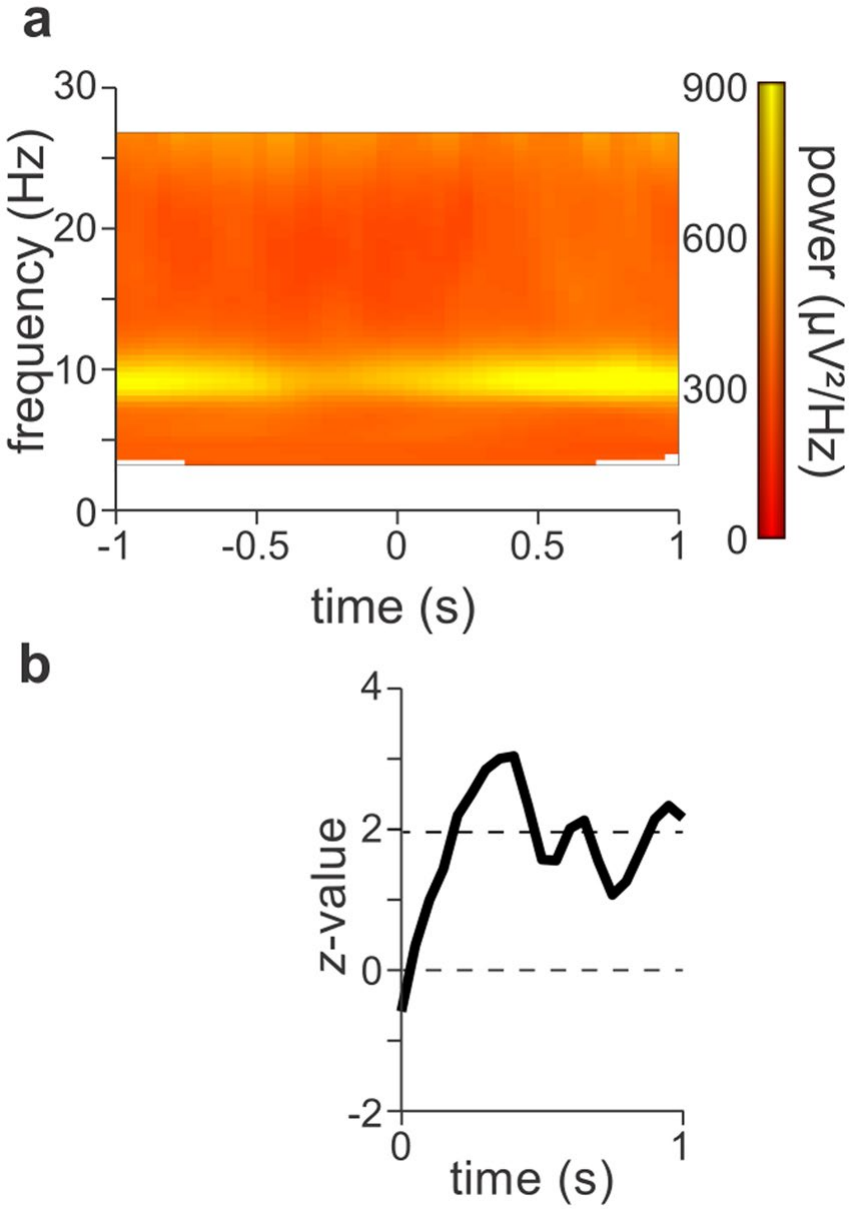
Alpha power predicts the fate of the subsequent perceptual duration right after the emergence of a percept. (**a**) TFR of the time window around the button press, averaged over the 14 parietal and occipital electrodes indicated in Fig. 2b. Alpha power decreases just before the perceptual reversal and increases thereafter (see Fig. S1 for a precise time course of alpha power only). (**b**) Alpha power after the onset of an individual perceptual representation was predictive of its subsequent duration, quantified by the *z*-values computed on a 500 ms long sliding window in the interval between 0 s and 1 s. The dotted line at 1.96 indicates the *z*-value threshold at *p*-value = 0.05.

We next addressed, with a finer time resolution, how soon after the emergence of each individual percept alpha power predicted its perceptual duration. We confirmed the observation that the largest changes during the perceptual reversal occurred in the alpha frequency band (Fig. 4a and Supplementary Fig. S1)^35–38^, which might be linked to transient saccades^39^ in response to the reorganization of the brain network^31^. We therefore concentrated on alpha power, which was calculated with a forward sliding windows (500 ms width, 50 ms step size) from the emergence until the end of each percept. We correlated the amount of alpha power within each 500 ms window with the duration of the subsequent dominant perceptual representation. We found that the linear correlation in the window centered at 400 ms after the report of the onset of the dominant percept was already highly significant (*z*-value = 3.286, *p*-value < 0.001, Fig. 4b).

**Figure 5:**
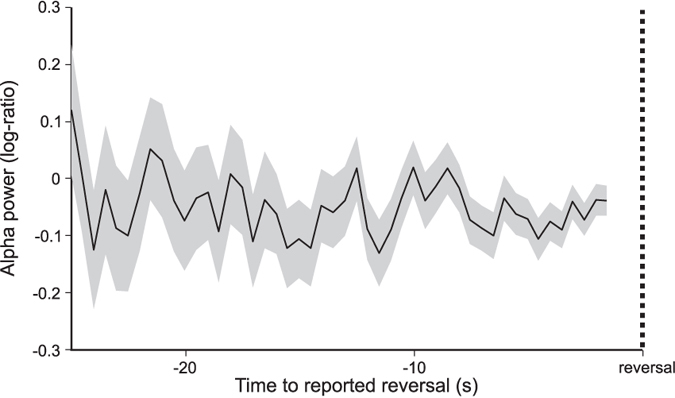
The time course of alpha power during the individual perceptual representations was stable during the period prior to a perceptual reversal. Values on the y-axis indicate the log of the ratio between alpha power on a sliding 1 s long window and alpha power in the interval of 1–2 s before the beginning of the individual perceptual representation. Gray areas indicate the standard error of the mean.

We then investigated whether alpha power declined or remained constant during the individual perceptual durations. Alpha power was calculated over sliding windows of 1 s long and 50% overlap, from the emergence to the end of each percept, excluding the 2 s interval around the button press. As shown in Fig. 5, alpha power remained constant during the individual perceptual duration before the perceptual reversal.

**Figure 6:**
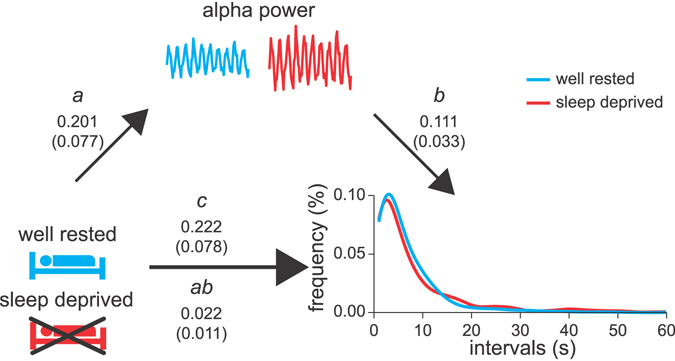
Alpha power partly mediates sleep deprivation-induced increased perceptual duration of perceptual representations. The estimated values are given for each path, with the s.e. shown in brackets. Sleep deprivation increased the duration of individual perceptual representations (path *c*). In particular, after sleep deprivation, the stability of individual perceptual representations was less likely to last less than 10 s and more likely to last around 20 s. In agreement with previous work, sleep deprivation increased eyes-open alpha power (path *a*). Mediation analysis suggested this increase in alpha power contribute to the increase in the duration of individual perceptual representations (path *ab*).

To evaluate whether experimental enhancement of eyes-open alpha power increased the duration of individual perceptual representations, participants were assessed, in balanced order, on a second day following sleep deprivation^40–43^. Sleep deprivation indeed affected the distribution of perceptual durations (Kolmogorov-Smirnov D = 0.089, *p*-value < 0.001) that became longer on average (0.251 log(s), s.e.m. 0.092, *z*-value = 2.728, *p*-value = 0.009). Mediation analysis was applied to evaluate whether the sleep deprivation-induced rightward shift in the distribution of perceptual durations could be attributed to the sleep deprivation-induced increase in alpha power during the individual percepts (0.202 1/log(µV^2^), s.e.m. 0.077, *z*-value 2.607, *p*-value = 0.009). Mediation was investigated using a linear mixed-effects model implementation of the Sobel test^44–46^. Following the terminology in mediation analysis literature^47^, the *a* path corresponds to the effect of sleep deprivation on alpha power, the *b* path is the effect of alpha power on the duration of individual perceptual representations adjusted for the effect of sleep deprivation, the *c* path is the unmediated effect of sleep deprivation on the duration of individual perceptual representations and *ab* is the part of the effect of sleep deprivation on the duration of individual perceptual representations that is mediated by its effect on alpha power (Fig. 6). Indeed, the effect of sleep deprivation on the duration of individual perceptual representations was in part mediated by its effect on alpha power (path *ab*, estimate 0.022, s.e.m. 0.011, *z*-value = 2.059, *p*-value = 0.039; estimate for the *a* path 0.201, s.e.m. 0.077; estimate for the *b* path 0.111, s.e.m. 0.033) while the unmediated effect (path *c*) was 0.222 (s.e.m. 0.078).

## Discussion

The present study addresses the role of alpha oscillations in adjusting the flexibility of the brain to either maintain its current configuration of neuronal activity and the corresponding perceptual representation, or rather switch to a different one. In a bistable perception paradigm, higher alpha power correlates with longer duration of the individual perceptual representations. The duration of the interval an individual perceptual representation will remain dominant for can already be predicted from the amount of alpha power observed during the very first second after the emergence of that representation. Intriguingly, the relationship between alpha power and perceptual duration holds even for perceptual representations lasting up to a minute, adding support to previous reports of long-range temporal determination in human brain oscillations with relevance to perception and behavior^13–16, 48^. The level of alpha power remains stable in the period between reversals, indicating that the association between alpha power and the stability of an individual perceptual representation persists throughout the perceptual duration. We found that, instead of being a spatially unspecific effects, the estimated generators of the alpha oscillations controlling perceptual stability were circumscribed and located in the high-level visual cortex, suggesting that the influence of alpha oscillations operates at a high level in the visual hierarchy. To substantiate functional involvement of alpha activity, we enhanced it by means of sleep deprivation. Indeed, sleep deprivation increased the average duration of an individual perceptual representation and this effect was in part mediated by the concurrently induced increase in alpha power.

The strong association between alpha power and perceptual stability found in our study builds on previous work linking spontaneous fluctuations in alpha power with trial-by-trial variability in cognitive performance^49–51^. In particular, alpha power is high just before or during trials with poorer performance in visual detection tasks^9–12^. These observations have been used to support the interpretation that alpha reflects the inhibition of the processing of visual information in lower visual areas, before the information reaches cortical regions involved in conscious perception and in the initiation of a behavioral response^10, 52^. According to this interpretation, a beneficial effect of this system is that alpha acts as a filter that prevents the broadcasting of irrelevant information, such as unimportant object’s attributes or distractors^12, 23, 53, 54^.

Our findings indicate that alpha oscillations operate a higher level of the cortical hierarchy and that their amplitude remains relatively stable during a perceptual duration. Based on these studies and the present work, we propose that the role of alpha oscillations is not to purely inhibit cortical activity but to stabilize the current configuration of neuronal activity and its corresponding perceptual representation. According to this interpretation, alpha power reflects the degree of locking of the neurodynamical attractor state of a network, which determines both its resilience to changes in input and its output predictability. Therefore, large alpha amplitudes in a given region primarily index the locking of the dynamical output that the region generates. The locking of the neurodynamical process might be implemented by a variety of underlying mechanisms: a controlled access to relevant input^55^, selection of neural object representations^8^, or the temporary inhibition of competing neural populations^11^. A locked neurodynamical attractor state supports ‘maintenance of the status quo’, a functional property that has previously also been proposed for beta oscillations^56^. Interestingly, Fig. 4a also shows a decrease in the beta band (15–20 Hz) in addition to the alpha band, suggesting that the reversal of bistable images involves a similar mechanism for both alpha and beta rhythms^37^.

Such stabilization would provide a parsimonious concept that could account for a range of diverging findings, including the observations that high alpha power may render the brain less responsive to incoming stimuli^6, 32^, support working memory by blocking interfering aspects^8, 57^, and inhibit irrelevant activity^12, 30, 54, 58^. In selective attention paradigms, the observed functional inhibition does not necessarily have to imply inhibition of activity in task-irrelevant areas, but can equally well result from strong resonating activity that is not easily perturbed by input. In memory paradigms, the same fixed dynamical output could represent the content to be memorized. For both paradigms, it is conceivable that this output could even support task-relevant neuronal ensembles, by providing them with predictable input rather than no input. ‘Task-irrelevant’ areas that show alpha need not be ‘turned off’, but may rather actively contribute to targeted processing in task-relevant areas by providing them with predictable input that could help overrule unpredictable distracting input.

The enhancement of alpha power by means of sleep deprivation increased the duration of perceptual representations. This observation provides a new view on the enigmatic mechanisms involved in the effects of sleep deprivation on sustained attention and cognitive switching. Sleep deprivation severely compromises sustained attention^59, 60^. Sleep-deprived subjects often fail to report the presence or change in appearance of the target stimulus^61, 62^ and the neuronal signature may lack traces of higher-order stimulus processing^63^. In addition to compromised sustained attention, cognitive inflexibility and perseveration are among the most consistently reported, yet poorly understood, cognitive sequelae of sleep deprivation^64–70^. The network locking hypothesis suggests that a sleep deprivation-induced increase in alpha power adversely affects the switching to brain states required to initiate stimulus evaluation and behavioral responding. The network locking hypothesis also explains the observation that sleep deprivation does not hinder, and may even promote, those executive functions that profit from enhanced perseveration^71, 72^.

Thus, in addition to its value for understanding the functional relevance of alpha oscillations, the network locking framework moreover promises a better understanding of the mechanisms involved in the sometimes devastating effects of insufficient sleep^73^.

## Methods

### Participants

Eight healthy adults (five male, age range 20–26 years, all right-handed), naive to the research question, participated in the experiment after giving informed written consent. The study protocol was approved by the medical ethics committee of the Academic Medical Centre of the University of Amsterdam according to the declaration of Helsinki and the experiments were performed in accordance with the relevant guidelines and regulations. All participants met the following criteria: (I) no self-reported sleep complaints, assessed using standard questionnaires^74–76^; (II) non-smoking; (III) no use of medication, including hormonal contraceptives; (IV) no neurological or psychiatric disorders. All had normal or corrected-to-normal vision, followed a regular sleep wake rhythm assessed by actigraphy (Actiwatch, Cambridge Neuro-Technology Ltd., Cambridge, UK) during the week before the experiments and refrained from caffeine and alcohol on the day before and during the experiment.

### Protocol

Prior to the assessment days, subjects either had a night of normal sleep or a night of total sleep deprivation. The order of the conditions was randomized and counterbalanced across participants. Successful completion of total sleep deprivation was verified using actigraphy. This verification is based on visual inspection of the cumulative distribution of immobility bout durations to exclude immobile period longer than 10 minutes, which might correspond to periods of sleep. All the analyses, except those assessing the effect of sleep deprivation on bistable perception, were conducted on the recordings acquired after normal sleep.

At five times during the day (at 10:30, 12:00, 13:30, 15:00 and 16:30 hr), participants viewed a static image of the Necker cube for 4 minutes (Fig. 1a). Participants were instructed to fixate on the center of the screen and to report perceptual reversals by pressing one of two buttons on a keyboard, where each button was associated with one of the two perceptual representations. The task was programmed in E-Prime 1.1 (Psychology Software Tools, Pittsburgh, PA). The duration of each individual perceptual representation was defined as the period between two subsequent button presses. Exceptionally short and long perceptual durations lasting less than 1.5 s or more than 60 s were excluded from the analysis. The probability distribution of duration of the individual perceptual representation was computed in intervals of 1 s and averaged across participants. Throughout the task, EEG was recorded at 1024 Hz, with a Micromed SD-LTM64 recorder (Micromed, Mogliano Veneto, Italy), using a 61-equidistant channel EEG cap (M10, Easycap, Munich, Germany), referenced at Cz. Impedance was kept below 10 kΩ.

### EEG Preprocessing

All EEG analyses were conducted using FieldTrip^77^, in Matlab 7.13 (MathWorks, Natick, MA). Noisy channels were interpolated using linear interpolation from the recordings of the neighboring channels for the scalp-level analysis and were not used for the source-reconstruction analysis. The signal was offline re-referenced to the average reference. Eye-blinks, movement artifacts, and other artifacts were corrected by rejecting noisy independent components^78^. Artifactual components were automatically identified using various statistical criteria that considered both the temporal and spatial characteristics of the artifacts^79^. EEG data were high-pass filtered at 0.5 Hz cutoff frequency (4th-order Butterworth filter) and notch-filtered at 50 Hz.

### Quantification Of Average Alpha Power During Individual Perceptual Representations

For the duration of each individual perceptual representation, EEG recordings were segmented in 1 s long, 50% overlapping time windows, starting at 1 s after the button press and ending, depending on the residual length of the segmentation, between 1.5 s and 1 s before the following button press. The power spectrum in the frequency range between 1 Hz and 30 Hz was estimated with the Welch’s method: the fast Fourier transform was computed on each 1 s long time window, after multiplication with a Hanning window, and averaged over 14 parietal and occipital electrodes and over the time windows belonging to each individual perceptual representation. The frequency band for alpha oscillations in our study was defined based on the observed peak in the power spectrum at 7–11 Hz (see Results).

### Regression Of The Duration of Perceptual Representations On Alpha Power

The first research question we tested was whether the duration of each individual representation was correlated with alpha power during that representation. Alpha power, computed on the 1 s long, 50% overlapping time windows as described above, was averaged for each of the stable perceptual representation intervals of variable duration, from 1 s after its onset to 1.5–1 s before the following button press, depending on the residual length of the segmentation. Both alpha power and the durations of individual perceptual representations were log-transformed. This interval of at least 2 s surrounding the reported onset of a representation was excluded from the analysis to prevent the confounding influence of the temporary decrease in alpha power thought to be due to the change in perceptual state^35–38^.

Alpha power’s predictive value of the duration of individual perceptual representations was evaluated using a linear mixed-effects regression model (LMEM), partitioning the otherwise correlated error terms across the four nested levels (subject, day, session, and individual perceptual representations) and accounting for the variable number of epochs across sessions. Maximum likelihood estimated regression coefficient significance was evaluated using the Wald test. LMEM were computed using the ‘lme4’ package in R 2.14^80^.

### Source Analysis

To identify the local brain region that showed a significant correlation between alpha power and the duration of the individual perceptual representations, we performed a two-step analysis: first, alpha power was estimated in each voxel which was inside the brain using a frequency-domain beamformer^33^ and, then, we calculated the correlation between alpha power and the durations of the individual perceptual durations.

In detail, the beamformer was applied on each voxel on a 1 cm spaced grid inside the brain of the template brain image. The frequency-domain beamformer is a spatial filter defined by the volume conduction model, cross-spectral density (CSD), and a regularization parameter^33^. The volume conduction model was calculated from a template MRI, distributed with SPM8^81^ and segmented using a 3-level boundary element model (BEM)^82^, each consisting of 1500 vertices. The conductivity for the three compartments (scalp, skull, and brain) was 0.33, 0.0041, and 0.33 S/m, respectively^83^. The CSD matrix was computed from the average power over the interval between two button presses, in the frequency band centered at 9 Hz with ± 2 Hz frequency smoothing with multitapers^34^ and the regularization parameter was kept at 5%. All the channels, except those that were marked as containing artifacts, were used for the computation of the CSD. To obtain a robust estimate of the CSD matrix, we applied a common filter approach, by which the CSD (and therefore the spatial filter) is calculated from all the individual perceptual representations in a session and then the spatial filter is reused on the data from each individual perceptual representation. This procedure returns the estimated alpha activity at each voxel in the brain for each individual perceptual representation.

The log of the alpha power was used as regressor for the log of the durations of the individual perceptual representations, using an LMEM, with participants, sessions, and individual epochs of perceptual representations as nested levels. The *z*-value for each voxel in the brain, which reflects how well alpha power in that voxel was associated with the interval duration, is then projected onto the cortical surface for illustration purposes.

### Time-Frequency Analysis Around the Perceptual Reversals

To investigate whether changes in any frequency band up to 30 Hz was modulated by the occurrence of the reversal of the perceptual representations, we calculated the time-frequency representation (TFR) of the power in the interval between −1 s and 1 s, centered on the button press. A fast Fourier transform at each frequency f_0_ between 4 Hz and 30 Hz was computed on sliding time windows whose duration was equal to the lengths of 5 waves at f_0_ (at 4 Hz, the length of the time window was 1250 ms and at 30 Hz, it was 167 ms) after multiplication with a Hanning taper.

### Alpha Power Predicts The Duration Of Individual Perceptual Representations

As we observed the largest changes in the alpha band around the time of the reversal (see Results), we asked the question of how soon after the onset of each individual perceptual representation alpha determined the fate of its duration. To increase the temporal resolution of the correlation after the onset of the perceptual representation, we calculated alpha power on sliding windows with shorter duration (500 ms) and larger overlap (50 ms step) starting at the reported onset of each individual perceptual representation. For each of the windows, LMEM as described above was applied to evaluate how well its log-transformed alpha power predicted the log-transformed duration of the individual perceptual representation interval it belonged to.

### Fluctuations In Alpha Power Between the Reversals

We then investigated whether alpha power after the perceptual reversal remained stable, increased or decreased during each individual perceptual representations. The intervals during which a perceptual representation was dominant were divided in 1 s long, 50% overlapping segments, excluding the 2 s around the button press. The time stamp was given by the midpoint of the segment, therefore the segments had time −1.5 s, −2 s, −2.5 s, etc. Alpha power was calculated on each segment and log-transformed. In order to normalize the level of alpha power, such that it was comparable across individual perceptual representations that had different durations, a baseline correction was applied by subtracting the amount of alpha power in the first available segment from the amount of alpha power in each segment. We then averaged over the segments that were at the same distance from the following reversal across all subjects. Because individual perceptual representations with longer durations were relative rare, to have robust estimates of the amount of alpha power at each time point we only considered segments that were within 25 s from the following reversal.

### Sleep Deprivation

A possible functional involvement of alpha power in the stability of a perceptual representation was furthermore experimentally addressed by manipulating alpha power by means of sleep deprivation, which is known to increase eyes-open alpha power^41^. As we observed higher alpha power associated with longer perceptual duration, we predicted that sleep deprivation-induced increase in alpha power will, on average, increase the duration of individual perceptual representations. This hypothesis relies on mediation analysis and can therefore be structured in three questions: 1) whether sleep deprivation increases alpha power; 2) whether sleep deprivation increases the duration of perceptual representations; 3) whether the increase in alpha power due to sleep deprivation mediates the increase in perceptual duration.

### Sleep Deprivation Effect On Alpha Power

The effect of sleep deprivation on alpha power, which was averaged over the parietal and occipital electrodes (indicated by markers in Fig. 2b), was computed using an LMEM, with the same nesting structure (subject-level, day-level, session-level, and at the level of individual epochs of perceptual representations). The Wald test was used to evaluate significance of the factor ‘sleep deprivation’ on log-transformed alpha power.

### Sleep Deprivation Effect On Perceptual Duration

The effect of sleep deprivation on the duration of individual perceptual representations was quantified using two separate tests: (I) whether the distribution of the durations of individual perceptual representations after normal sleep and after sleep deprivation were different was evaluated using a Kolmogorov-Smirnov test; (II) whether the average duration of individual perceptual representations was longer after sleep deprivation than after normal sleep, was evaluated using LMEM, with four nested levels and sleep deprivation as factor, on the log-transformed duration of the intervals between button presses.

### Alpha Power Mediation Of The Sleep Deprivation Effect On Perceptual Duration

The possible mediation by alpha power of the effect of sleep deprivation on the average duration of individual perceptual representations was evaluated using an LMEM implementation of the Sobel test^44–46^. Regression coefficient estimates and their standard errors were computed for each path defined in Fig. 6 using an LMEM with a nested structure as above (subject-level, day-level, session-level, and at the level of individual epochs of perceptual representations). The unmediated path *c* represents the relation of the individual perceptual representations (dependent variable) to sleep deprivation (independent variable). Path *a* represents the relation of the mediatior, in this case alpha power, (dependent variable) to sleep deprivation (independent variable). Path *b* represents the relation of the individual perceptual representations (dependent variable) to the mediator, i.e. alpha power, adjusted for sleep deprivation (both alpha power and sleep deprivation were the independent variables in the model, but *b* represents only the estimates of alpha power on the dependent variable). The Sobel test is defined as the ratio between the estimated *ab* path and its standard error. The former is computed as the product of *a* and *b* and the latter as √(a^2^σ_b_^2^ + b^2^σ_a_^2^)^84^. The ratio yields a *z*-value that can be used to obtain a *p*-value.

The complete code in Matlab and R can be downloaded from *https://github.com/gpiantoni/neckersd*.

## Electronic supplementary material

Supplementary Material
