# Supplementary material for: Alpha Power Predicts Persistence of Bistable Perception
Source: Sci Rep. 2017 Jul 12;7:5208. doi: 10.1038/s41598-017-05610-8 (PMC5507912; doi:10.1038/s41598-017-05610-8)
Supplement: Supplementary file 1 — Supplementary Material [file 41598_2017_5610_MOESM1_ESM.pdf]

Supplementary Material to "Alpha Power Predicts Persistence of Bistable Perception"

G. Piantoni, N. Romeijn, G. Gomez-Herrero, Y. D. Van Der Werf, E. J. W. Van Someren

Figure S1

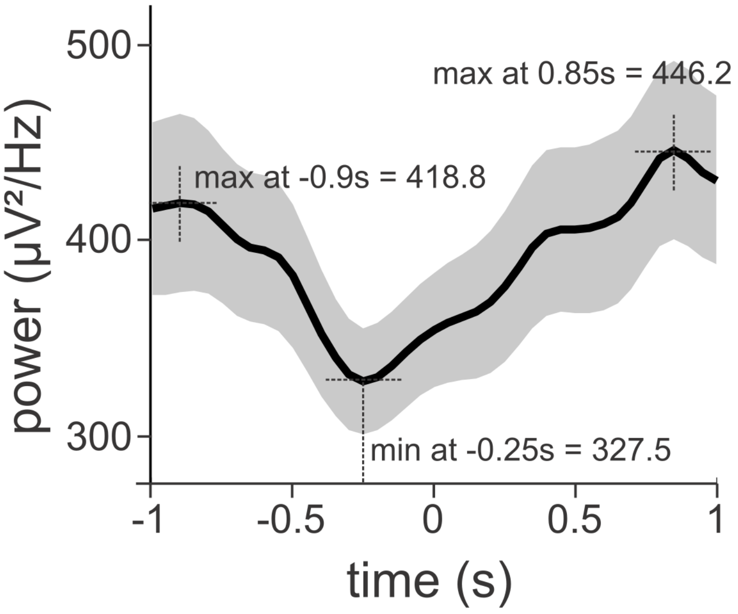

Alpha power decreases around the time of the perceptual reversal. Black line indicates the level of alpha power, averaged across subjects (gray area indicates the standard error of the mean), over the 2 s window around the button press indicating the perceptual reversal. Alpha power starts decreasing at around -900 ms before the report and reaches a minimum at -250 ms. This minimal value of alpha power is thought to correspond to the perceptual reversal, as it takes ~250 ms to report the changes in the bistable image[1–3]. After the perceptual reversal, alpha power rises again for a few hundreds ms before reaching its maximum in this window at 850 ms.

References

1. Luce, R. D. *Response Times: Their Role in Inferring Elementary Mental Organization*. 562 (Oxford University Press, 1986).

2. VanRullen, R., Reddy, L. & Koch, C. The continuous wagon wheel illusion is associated with changes in electroencephalogram power at approximately 13 Hz. *J. Neurosci.* **26,** 502–7 (2006).

3. Piantoni, G., Kline, K. A. & Eagleman, D. M. Beta oscillations correlate with the probability of perceiving rivalrous visual stimuli. *J. Vis.* **10,** 18 (2010).
